# Development and psychometric properties of knee-specific body-perception questionnaire in people with knee osteoarthritis: The Fremantle Knee Awareness Questionnaire

**DOI:** 10.1371/journal.pone.0179225

**Published:** 2017-06-26

**Authors:** Tomohiko Nishigami, Akira Mibu, Katsuyoshi Tanaka, Yuh Yamashita, Eiji Yamada, Benedict M. Wand, Mark J. Catley, Tasha R. Stanton, G. Lorimer Moseley

**Affiliations:** 1 Department of Nursing and Physical Therapy, Konan Woman’s University, Kobe, Hyogo, Japan; 2 Department of Rehabilitation, Tanabe Orthopaedics, Osaka, Osaka, Japan; 3 Department of Rehabilitation, Morinaga Orthopedic Clinic, Saga, Saga, Japan; 4 Joint Surgery Centre, Kaisei Hospital, Sakaide, Kagawa, Japan; 5 The School of Physiotherapy, The University of Notre Dame Australia, Fremantle, WA, Australia; 6 Sansom Institute for Health Research, University of South Australia, SA, Australia; 7 Neuroscience Research Australia, Sydney, NSW, Australia; Tokai Daigaku, JAPAN

## Abstract

**Background:**

Recent systematic reviews have demonstrated that pain associated with knee osteoarthritis (OA) is a complex phenomenon that involves various contributors. People with knee OA exhibit symptoms of impaired body-perception, including reduced tactile acuity, impairments in limb laterality recognition, and degraded proprioceptive acuity. The Fremantle Back Awareness Questionnaire (FreBAQ) was developed to assess body-perception specific to the back in people with chronic low back pain. The aim of this study was to develop and assess the psychometric properties of a knee-specific version of the FreBAQ-J (FreKAQ-J), determine whether people with knee pain experience perceptual impairments and investigate the relationship between disturbed self-perception and clinical status.

**Methods:**

Sixty-five people with knee OA completed the FreKAQ-J. A subset of the participants completed the FreKAQ-J again two-weeks later. Rasch analysis was used to assess item order, targeting, category ordering, unidimensionality, person fit, internal consistency, and differential item functioning. Validity was investigated by examining the relationship between the FreKAQ-J and clinical valuables.

**Results:**

The FreKAQ-J had acceptable internal consistency, unidimensionality, good test-retest reliability, and was functional on the category rating scale. The FreKAQ-J was significantly correlated with pain in motion, disability, pain-related catastrophizing, fear of movement, and anxiety symptomatology.

**Conclusions:**

We developed FreKAQ-J by modifying the FreBAQ-J. The FreKAQ-J fits the Rasch measurement model well and is suitable for use in people with knee OA. Altered body perception may be worth evaluating when managing people with knee OA.

## Introduction

Knee osteoarthritis (OA) is associated with a variety of symptoms, such as pain, stiffness and loss of function [1]. While structural abnormalities have been related to pain in people with knee OA, the relationship is modest [2] and such abnormalities are often observed in people without knee pain [3]. Recent systematic reviews have demonstrated that pain associated with knee OA is a complex phenomenon that involves various contributors, including lifestyle, cognitive factors and peripheral/central sensitization [4–7]. Similar to other chronic pain conditions, such as low back pain [8–13] and complex regional pain syndrome [14–16], people with chronic knee OA exhibit symptoms of impaired body-perception, including reduced tactile acuity [17], impairments in limb laterality recognition [18], and degraded proprioceptive acuity [19, 20]. Furthermore, neuroimaging studies of people with chronic OA demonstrate morphological and functional changes of cortical areas such as gray matter loss [21], disrupted whole-brain morphological structure [22], functional reorganization of the default mode network [23] and reorganization of the motor cortex [24]. To our knowledge, there has been no attempt to explicitly determine if altered self-perception is a feature of chronic knee OA or to investigate if altered self-perception is related to the severity of the clinical condition.

Recently, the Fremantle Back Awareness Questionnaire (FreBAQ) was developed to assess body-perception specific to the back in people with chronic low back pain [25] and it has since been translated into Japanese; the FreBAQ-J [26]. The FreBAQ is composed of nine items investigating neglect-like symptoms, reduced proprioceptive acuity, and issues of body shape and size. The FreBAQ score is associated with clinical variables including pain intensity, duration of pain, disability, and catastrophizing and appears to have acceptable psychometric properties [25–27]. We were interested in examining whether people with chronic knee OA also endorsed symptoms consistent with impaired self-perception and whether disturbed self-perception is related to clinical status. Therefore, the aims of this study were to:

1. Develop and assess the psychometric properties of a knee-specific version of the FreBAQ-J.
2. Determine whether people with chronic knee pain experience perceptual impairments.
3. Investigate the relationship between disturbed self-perception and clinical status.

## Methods

Ethical approval was obtained from the institutional ethics committee of Konan Woman’s University. Written informed consent was obtained from all subjects prior to the study. The study was conducted in compliance with the Declaration of Helsinki.

### Participants

Sixty-five people with knee OA were recruited consecutively from two orthopedic clinics and one Joint Surgery Center. All patients were screened and recruited by medical doctor. Inclusion criteria were a diagnosis of knee OA based on clinical guidelines (American College of Rheumatology) [28], knee pain for >3 months (as chronic pain is defined as persistent or recurrent pain lasting longer than 3 months), with a Kellgren-Lawrence (KL) score of 2 or more and age between 40 and 85 years. The exclusion criteria were total knee arthroplasty, serious pathologies (unhealed fractures, tumors, acute trauma, or serious illness), neurological findings (muscle weakness, loss of sensation or reflexes), and diagnosed psychiatric disorders. An equal number of healthy individuals with no history of knee OA and who matched the age and gender of the enrolled patients were recruited as controls.

### Development of the Fremantle Knee Awareness Questionnaire (FreKAQ-J)

The FreBAQ-J was initially developed using a forward–backward translation method [26]. To develop a knee specific version, the FreKAQ-J, the Japanese character for ‘knee’ was substituted for ‘back’. We deemed item 9 of the FreBAQ-J (My back feels lopsided) to be redundant as feelings of asymmetry do not apply to the knee. We adapted the item to reflect a perceptual difference between sides. The direct translation of item 9, from Japanese to English, was “My knee feels different with right and left (One side feels dull or fat)”.

### Procedure

Demographic data (age, gender, height, weight) were assessed in all participants, and pain duration, pain intensity, pain-related catastrophizing, fear of movement, anxiety, depressive symptomatology and knee pain-related disability were evaluated in the participants with knee OA. Pain intensity at rest and during movement were measured using a 0–100 visual analog scale anchored at left with “0 = no pain” and at right with “100 = unbearable pain” [29, 30, 31]. Pain-related catastrophizing was measured using the Japanese version of the Pain Catastrophizing Scale (PCS) [32, 33] and pain-related fear was assessed using the Japanese version of the Tampa Scale of Kinesiophobia (TSK) [34, 35]. We evaluated anxiety and depression with the Japanese version of the Hospital Anxiety and Depression Scale (HADS), which consists of seven anxiety and seven depression items, from which separate anxiety and depression scores are calculated [36–38]. Functional disability was measured using the Japanese-validated version of Oxford Knee Score (OKS) (0–48; higher scores indicate more functional ability) for patients with knee OA [39, 40]. In addition, all of the participants completed the FreKAQ-J. The instructions used for the control participants when filling out the FreKAQ-J read ‘please indicate the degree to which your knee feels this way today’.

### Psychometric assessment of the FreKAQ-J

Only participants with knee OA was used for Rasch analysis. We used Rasch analysis to analyze the psychometric properties of the FreKAQ-J, following the same procedure used in our previous study [26]. Rasch analysis allows us to compare the ordinal FreKAQ-J data to a probabilistic mathematical model that is based on fundamental principles of measurement [41–43]. Applied here, the model assumes that a person with comparatively frequent perceptual impairment is more likely to endorse any of the FreKAQ-J items and the likelihood of an item that reflects comparatively little perceptual impairment is more likely to be endorsed by everyone. We used Winsteps software (v3.90.2, Chicago, Illinois) to analyze the following:

#### Item order

While all the FreKAQ-J items share the same Likert scale, they are not endorsed to the same extent by the sample. That is, some items are relatively harder to endorse because they reflect experiences that are more likely to be perceived by only those with high perceptual impairment. Recent evaluations of the FreBAQ [27] found the items differed in regard to endorsability and the hierarchal order of the items supported the underlying theoretical construct of perceptual impairment, thus providing evidence of validity [27]. Here, we assessed the item order through an analysis of the relative endorsability measures of the items.

#### Differential test functioning

Differential test functioning could assess whether knee OA patients using the FreKAQ-J and chronic low back pain patients using the FreBAQ-J [26] is a similar manner. Differential test functioning was explored if an item yielded a significant difference of greater than 0.5 logits between samples.

#### Targeting

Targeting refers to how well the FreKAQ-J items targeted the participants. Rasch analysis is a probabilistic model that, in this instance, assumes people with greater perceptual disturbance will be more agreeable with the FreKAQ-J items than those with lesser perceptual disturbance and items indicative of greater disturbance are less endorsable than those indicative of lesser disturbance. Thus, a scale is considered to target the sample well when the average agreeability of the sample is similar to the average endorsiblity of the items with is anchored at 0 logits by the Rasch analysis software. We evaluated targeting by visual inspection of the distribution of person and item thresholds and consideration of the summary statistics. We also considered the presence of floor and ceiling effects.

#### Category order

We assessed category order to ensure the Likert scale functioned as expected. The FreKAQ-J has five response categories (0 = Never, 1 = Rarely, 2 = Occasionally, 3 = Often, 4 = Always). Category probability curves, and average measure and category fit statistics (infit and outfit) were used to explore rating scale functioning. If a disordered threshold is found, the rating scale collapses. Fit statistics are recommended to be between 0.6 and 1.4 [44, 45]. Moreover, in a well-functioning rating scale, each curve has a distinct peak and 4 clear thresholds that represent the point at which the likelihood of endorsing one category is equal to that of endorsing the next. Disordered thresholds can occur if a category is underutilized or respondents use the categories in an unexpected manner (eg, respondents cannot differentiate between the categories).

#### Unidimensionality

We intend to summate the FreKAQ-J to provide an overall measure of perceptual impairment. Individual items should share in common this unidimensional construct yet be sufficiently different to warrant their inclusion. Assessment of fit evaluates unidimensionality by identifying items that function unexpectedly and the principal component analysis of residuals (PCA) identifies unexpected clusters of items suggestive of a secondary dimension that could threaten measurement of the primary dimension. Excessively large fit residuals (>1.4 logits) indicate a large difference between the expected and observed performance of an item [46] and may indicate that the item is assessing a construct other than the intended construct. Fit statistics are chi-square based and are reported as mean-squares (in logits), with an expected value of 1 logit. Excessively small fit residuals (<0.6 logits) identify items that behave too predictably [46]. We compared both infit (information-weighted) and outfit (outlier-sensitive) statistics and inspected the item characteristic curves of misfitting items to determine how they behaved for participants of differing agreeability. The PCA residual correlation matrix was visually inspected to identify clusters of items that would be suggestive of a second dimension. An eigenvalue greater than 2.0 for the PCA of residuals suggests a second dimension [47]. Response dependency between the items was examined by inspecting the residual correlation matrix [48] for pairs of items with correlations exceeding 0.4 [49, 50].

#### Person fit

Participants with outfit residuals greater than 1.5 logits were examined to determine the reason for poor fit [51]. They were compared with those who fit the model using Fisher’s exact test [52] of significance (for gender) or the Mann-Whitney U test (for age, pain intensity, pain duration disability and FreKAQ-J). Response strings of misfitting participants were analyzed to identify patterns in their responses.

#### Internal consistency

Winsteps provides a person reliability index and Cronbach’s alpha as indicators of internal consistency [53] and both should exceed 0.7 [48]. A minimum value of 0.7 is suggested for group use, and a minimum of 0.85 is suggested for individual use [48].

#### Differential item functioning (DIF)

Items should function in a similar manner for all people of similar levels of agreeability. We assessed for DIF across 5 subgroups: gender, age (≤65, >65 years), pain during motion (≤50, >50 mm), pain duration (≤1, >1 years) and disability (median split; ≤33, >33). DIF was tested using a Mantel–Haenszel chi-square test with significance set at p = 0.01 for each item. Item bias was explored if an item yielded a significant difference of greater than 0.5 logits between subgroups [54].

### Test-retest reliability

FreKAQ-J reliability was assessed using scores obtained from a second round of the questionnaire administered within 2 weeks of the first questionnaire completion. We only assessed test-retest reliability in outpatients to minimize the effect of treatment on clinical status. An intraclass correlation coefficient (ICC) 2-way mixed model with absolute agreement was used to determine measurement reliability. ICC values < 0.40 were considered to indicate poor reliability, 0.40–0.75 fair to good reliability, and 0.75–1.00 excellent reliability [55].

### Relationship to clinical status

Comparisons between the patients and the control group, and the correlation analysis were evaluated using the Statistical Package for Social Sciences Version 22.0 (IBM SPSS Statistics for MAC, Version 22.0. Armonk, NY: IBM Corp.). Data distribution was tested for homoscedasticity using the Kolmogorov-Smirnov test. Age, height, weight and FreKAQ-J (for discriminative validity) were compared between the knee OA group and the control group using Student t-test. Gender was compared between the knee OA group and the control group using Fisher exact test [52]. P values of less than 0.05 were considered statistically significant.

A series of univariate correlations was performed examining the relationships between the FreKAQ-J total raw ordinal level scores and: duration of pain, pain intensity, disability, pain catastrophizing, kinesiophobia, anxiety, and depression in the knee OA group. These correlations were investigated with Spearman’s correlation coefficient. We adjusted alpha to 0.007 because we undertook seven separate analyses.

## Results

All participants completed all aspects of this study. Both group characteristics are summarized in Table 1. Table 2 describes the frequency of responses for each item in the knee OA group.

**Table 1:** Demographic and clinical information. OKS: Oxford Knee Score, PCS: Pain Catastrophizing Scale, TSK: Tampa Scale of Kinesiophobia, HADS: Hospital Anxiety and Depression Scale.

Characteristics	Knee OA (n = 65)	Control (n = 65)
	Mean (SD) or N (%)	Mean (SD) or N (%)
Demographic information		
Gender (female)	50 (76.9%)	50 (76.9%)
Age (years)	68.5 (9.1)	66.7 (7.2)
Height (cm)	157.1 (7.1)	158.2 (7.6)
Weight (kg)	59.4 (10.1)	56.9 (9.2)
Body mass index	24.0 (3.5)	22.6 (2.6)
Clinical status		
Single or bilateral knee OA (single)	51 (78.4%)	
Duration of pain intensity (month)	57.7 (88.4)	
Pain intensity		
Rest	19.6 (21.7)	
Motion	43.5 (24.1)	
OKS	31.4 (9.8)	
Catastrophization (PCS)	24.4 (12.6)	
Kinesiophobia (TSK)	39.9 (5.7)	
Anxiety (HADS)	5.4 (3.1)	
Depression (HADS)	5.6 (2.6)	
FreKAQ-J	12.4 (7.6)	3.4 (4.4)

**Table 2:** Frequency of responses to each item of the FreKAQ-J.

Item	Never N(%)	Rarely N(%)	Occasionally N(%)	Often N(%)	Always N(%)	Median	Mean (SD)
1. I feel like my knee is not part of my own body	25 (38.5)	20 (30.8)	9 (13.8)	8 (12.3)	3 (4.6)	1	1.1 (1.1)
2. To move my knee the way I want to, I feel like I have to concentrate all my nerves there	14 (21.5)	18 (27.7)	13 (20.0)	11 (16.9)	9 (13.8)	2	1.7 (1.3)
3. Sometimes I feel like my knee moves without any connection to what I intend it to do	29 (44.6)	17 (26.2)	11 (16.9)	7 (10.8)	1 (1.5)	1	0.9 (1.0)
4. When performing activities of daily living (housework, work, etc.), I do not know how much my knee is moving	25 (38.5)	19 (29.2)	10 (15.4)	9 (13.8)	2 (3.1)	1	1.1 (1.1)
5. When performing activities of daily living (housework, work, etc.), I do not know what kind of position my knee is in	22 (35.4)	16 (24.6)	14 (21.5)	10 (15.4)	2 (3.1)	1	1.2 (1.1)
6. I cannot image my knee’s contour correctly	18 (27.7)	19 (29.2)	11 (16.9)	17 (26.2)	0 (0.0)	1	1.4 (1.1)
7. I feel like my knee is bigger (swollen)	19 (29.2)	12 (18.5)	19 (29.2)	7 (10.8)	8 (12.3)	2	1.5 (1.3)
8. I feel like my knee has shrunk	31 (47.7)	18 (27.7)	10 (15.4)	5 (7.7)	1 (1.5)	1	0.8 (1.0)
9. My knee feels differences with right and left. (One side feels dull or fat)	10 (15.4)	6 (9.2)	16 (24.6)	21 (32.3)	12 (18.5)	3	2.2 (1.2)
Total						12	12.4 (7.6)

### Psychometric assessment of the FreKAQ-J

#### Item order

**Fig 1:**
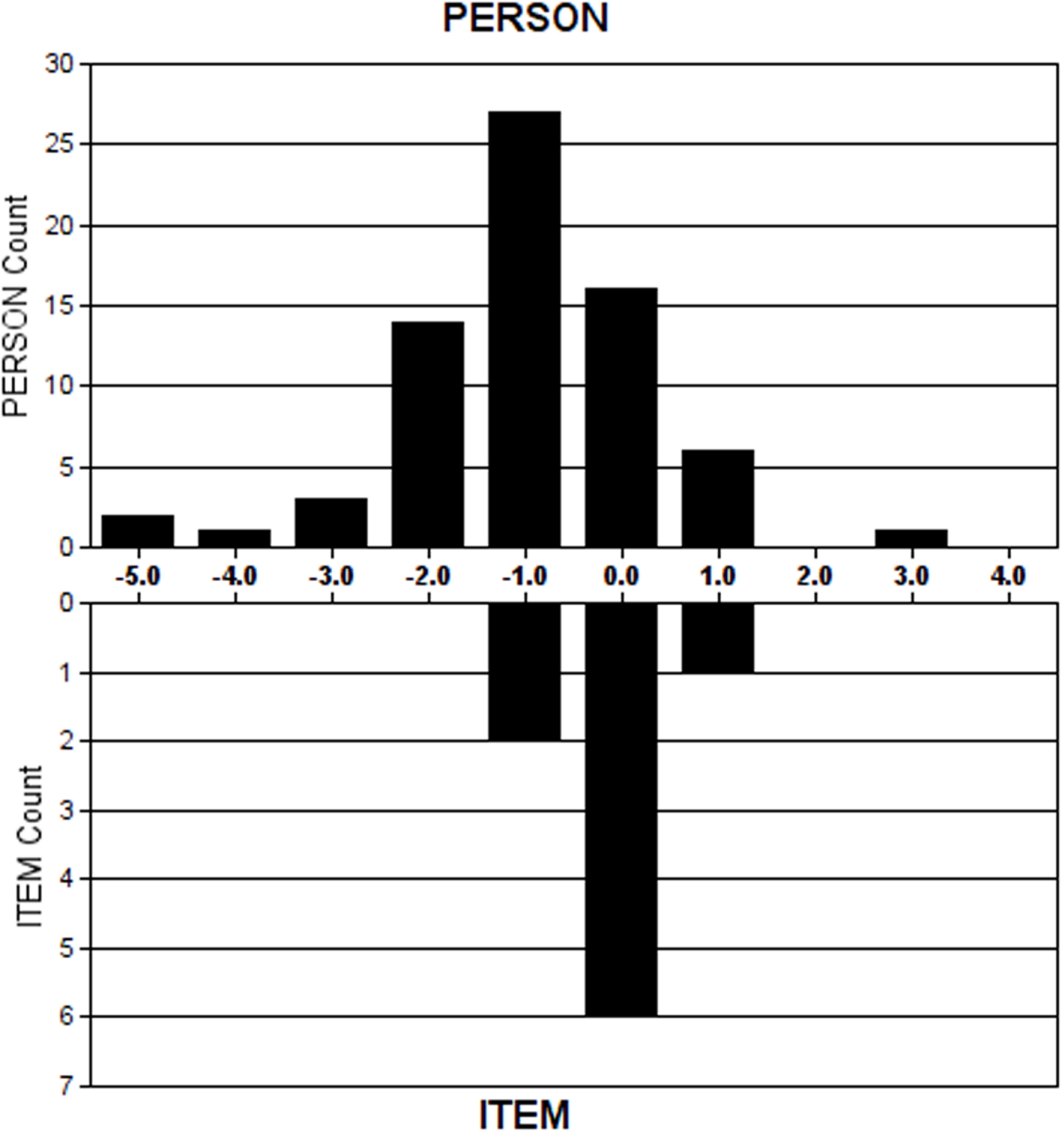
Item-person threshold map. Persons of lesser disturbed body perception and items easier to endorse are located on the left side of the logit scale (ie, < 0 logits); Persons of higher disturbed body perception and items of greater difficulty to endorse are located to the right of the logit scale (ie, > 0 logits). Item endorsability mean is set at 0 logits by default.

Fig 1 shows the item ordering and the relationship between FreKAQ-J items and person logit ratings. The item ordering is also evident from the item endorsability thresholds presented in Table 3.

**Table 3:** Average item endorsability thresholds, including fit statistics.

Item	Measure (Logits)	SE	Infit (mnsq)	Outfit (mnsq)
9	-1.21	0.14	**1.46**	**1.41**
2	-0.49	0.14	0.88	0.85
7	-0.29	0.14	**1.74**	**1.61**
6	-0.07	0.14	0.66	0.67
5	0.14	0.15	0.82	0.81
1	0.32	0.15	1.16	1.38
4	0.32	0.15	**0.56**	**0.53**
3	0.56	0.16	0.72	0.71
8	0.73	0.16	1.09	0.87

#### Differential test functioning

**Fig 2:**
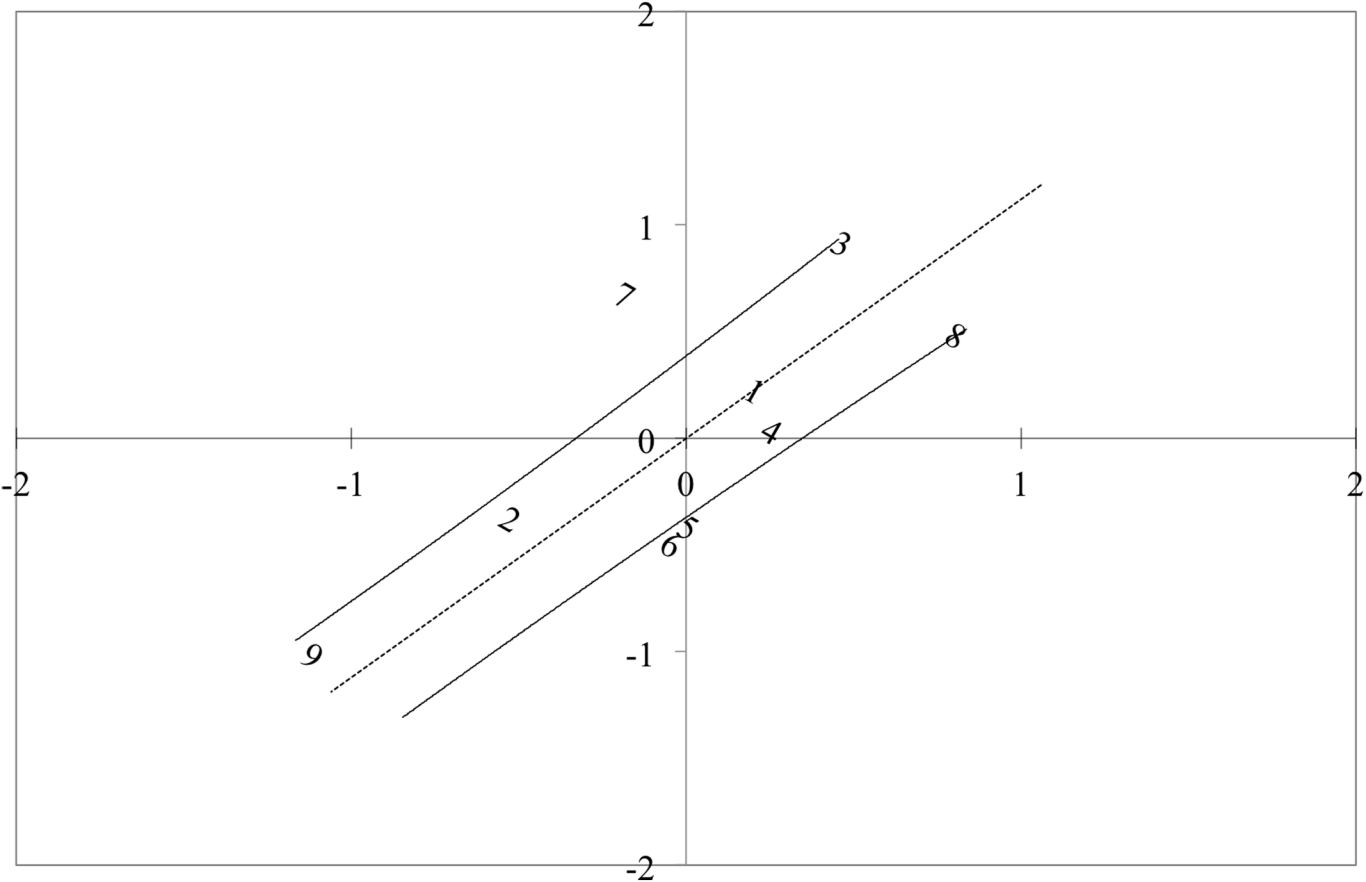
Differential test functioning between the FreBAQ-J and FreKAQ-J. The dashed line shows a trend-line through the mean of both sets of items, and the black lines show the upper and lower 95% confidence bands.

The differential test functioning results (Fig 2) supported this finding by showing knee OA patients used the FreKAQ-J in a similar manner to chronic low back pain patients using the FreBAQ-J. Items at the extremities of the scale were stable with some disordering in the middle items.

#### Targeting

Participants with low levels of disturbed body perception were not targeted well by the FreKAQ-J. The average person endorsability was -0.92 logits (standard deviation (SD) 1.34, range -3.61 to 3.12) whereas default item endorsability average = 0 logits (SD 0.57, range -1.21 to 0.73). That person agreeability was shifted to the left when compared with item endorsability indicates that the questionnaire better targets those with higher levels of disturbance than those with lower levels of disturbance. Item 9 was the easiest item for participants to endorse. Item 8 was the most difficult item to endorse. Only two participants (3.0%) scored zero for all items; no participants scored full points on all items.

#### Category order

**Fig 3:**
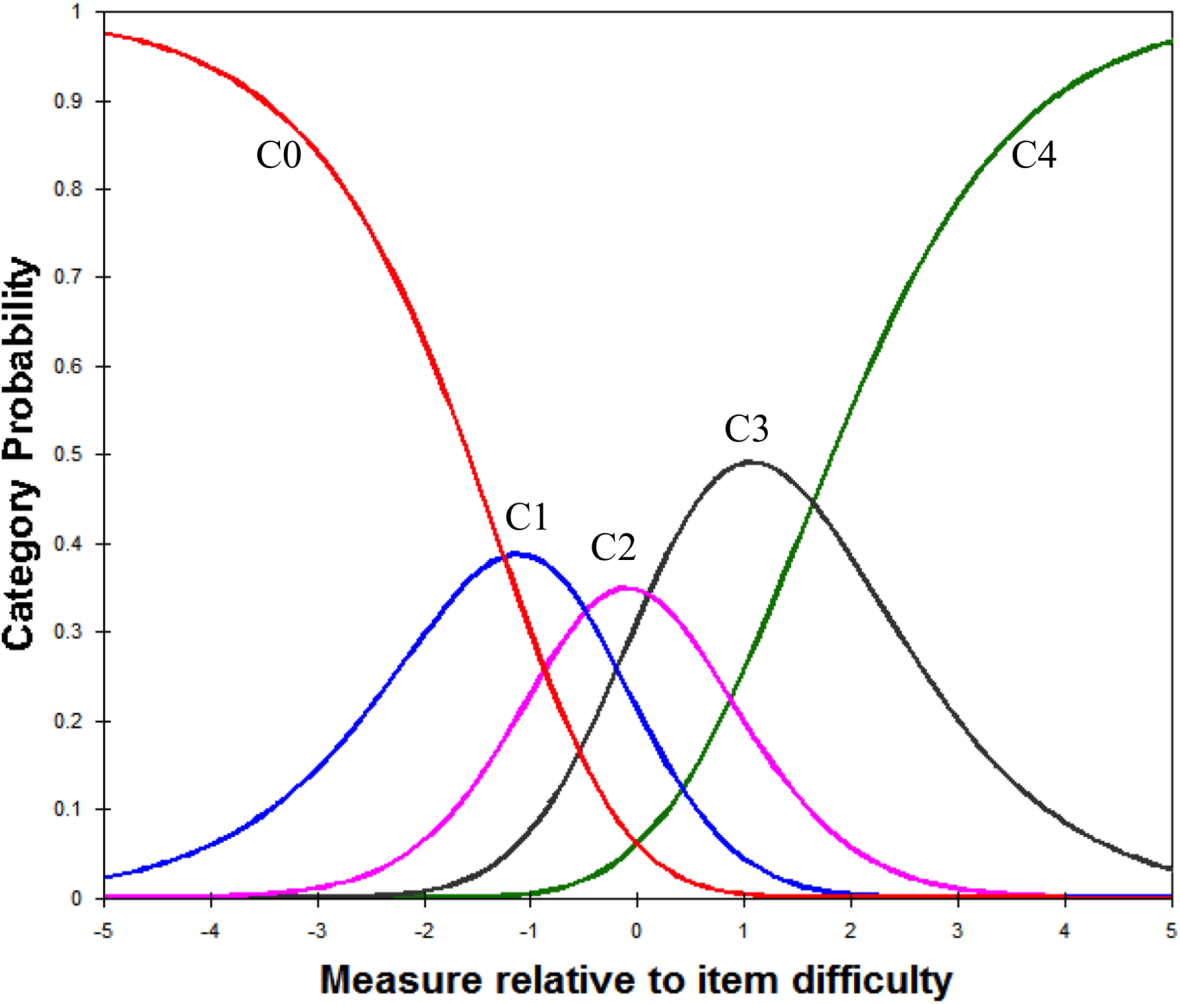
Probability curves for the 5-category Japanese version of the Fremantle Knee Awareness Questionnaire. C0, never; C1, rarely; C2, occasionally; C3, sometimes; C4, always).

The average agreeability measures of the respondents advanced as expected across the rating scale categories and there was neither excessive positive nor negative fit statistics, suggesting the category structure is adequate (Table 4). Each category has a distinct peak suggesting the categories are not disordered—that is, the step calibrations (the thresholds between categories) are ordered as expected (Fig 3).

**Table 4:** Average category score thresholds, including fit statistics.

Category score	Measure (Logits)	Infit	Outfit
0	-1.86	1.11	1.13
1	-1.10	0.82	0.83
2	-0.33	0.79	0.76
3	0.39	1.02	0.95
4	1.15	1.32	1.28

#### Unidimensionality

Table 3 summarizes the fit statistics for the nine items. Two items (Item 7 and 9) demonstrated excessive positive infit and outfit statistics and one item (Item 4) demonstrated slightly excessive negative infit and outfit statistics. Critically however, these results do not pose a threat to validity. PCA of residuals indicated that the unexplained variance of the first contrast was 1.9 eigenvalue units, indicating unidimensionality. Assessment of local dependence identified no strong correlations between all items. This suggested that the responses to all items were independent.

#### Person fit

Analysis of person fit identified 13 participants (20.0%) with excessive positive outfit (>1.5 logits). No significant associations were found between between those who fit versus those who did not fit the Rasch model: age (p = 0.13) gender (p = 0.15), pain intensity at rest (p = 0.60), pain during motion (p = 0.90), pain duration (p = 0.15), disability (p = 0.34), and the FreKAQ-J score (p = 0.80).

#### Internal consistency

The person reliability was 0.81 and the Cronbach’s alpha was 0.88 suggesting the FreKAQ-J has good internal consistency and is suitable for both individual and group use.

#### Differential item functioning (DIF)

Differential item functioning, characterized as a significant difference of greater than 0.5 logits, was not observed between age, gender, pain intensity, pain duration or disability subgroups suggesting the items were not biased.

### Test-retest reliability

Of the participants who reported no change (the difference between first and second round of pain intensity during movement is under 10 mm) in pain intensity during the past 2 weeks (n = 23), there was excellent agreement between test and retest total scores, with an ICC_3,1_ of 0.76 [95% confidence interval (CI) 0.52–0.89].

### Relationship to clinical status

There was no significant difference in age (p = 0.21), gender (p = 1.0), height (p = 0.36) or weight (p = 0.14) between the knee OA group and the control group. The knee OA group scored significantly higher on the FreKAQ-J than the control group (p < 0.001, mean difference = 9.0, 95%CI = 6.7 to 11.1). The FreKAQ-J was significantly correlated with, pain during motion (rho = 0.37), OKS (rho = -0.41), PCS (rho = 0.70) and TSK (rho = 0.49) and anxiety (rho = 0.46; p < 0.007 for all), but not duration of pain intensity (rho = -0.06; p = 0.76), pain at rest (rho = 0.27; p = 0.02), and depression (rho = 0.32; p = 0.01) (Table 5).

**Table 5:** Correlations between the FreKAQ-J total score and clinical symptom.

	Correlation coefficient (R)	p value
Duration of pain intensity	-0.06	0.76
Pain intensity		
Rest	0.27	0.02
Motion	0.37	**0.002**
Disability		
OKS	-0.41	**0.001**
Catastrophization (PCS)	0.70	**< 0.001**
Kinesiophobia (TSK)	0.49	**< 0.001**
Anxiety	0.46	**< 0.001**
Depression	0.32	0.01

## Discussion

We aimed to develop a knee-specific Japanese version of the FreBAQ, assess its psychometric properties, determine whether people with knee OA have self-reported perceptual impairment and investigate the relationship between impaired self-perception and clinical status. Our results suggest that the newly developed FreKAQ-J has acceptable construct validity, internal consistency, and test-retest reliability. That the nine items form a unidimensional scale suggests the score can be summed to provide a measure of perceptual impairment. The scale is not biased by other characteristics, such as age or gender, and the category rating scale functions as expected. Only two people with knee OA (3.0%) scored zero and none had a full score, suggesting that the FreKAQ-J has neither floor nor ceiling effects, although those with very minor perceptual disturbances are less well captured by the FreKAQ-J than the rest. Participants with knee OA scored higher than healthy control. Overall, the FreKAQ-J showed adequate psychometric properties for use in evaluating impaired body perception of people with knee OA in the Japanese population.

The differential test functioning results between the FreKAQ-J and the FreBAQ-J demonstrated that items at the top and bottom of the scale were stable with some disordering in the middle items, suggesting the construct between the FreKAQ-J and the FreBAQ-J is similar. The FreBAQ has a theoretical construct of perceptual impairment [27], therefore, the FreKAQ-J is also likely a valid assessment of perceptual impairment.

Rating categories complied with the set criteria for category functioning, and step measures endorsed monotonically from easy to hard across category responses as was seen for the FreBAQ-J [26], supporting proper category order.

The internal consistency of the FreKAQ-J was good (person reliability index of 0.81) and aligned with that of the FreBAQ-J in patients with low back pain (person reliability index of 0.76) [26]. Also, the ICC score was ICC of 0.76 (95%CI 0.52–0.89), indicating the FreKAQ-J had excellent reliability–again corroborating the earlier work on the FreBAQ-J in patients with low back pain (0.81, 95% CI 0.67–0.89) [26]. To explore the reason for the slightly lower reliability of the FreKAQ-J in comparison to the FreBAQ-J we calculated ICCs of individual items in both scales. One item in the FreBAQ-J (Item 6) had low test-retest reliability (< 0.40), whereas three items in FreKAQ-J (Item1, 4, 5) had low test-retest reliability (< 0.40). This may impact on difference of the reliability of the FreKAQ-J and FreBAQ-J.

No significant difference in participants with and without misfting was found. In the FreBAQ-J, misfitting participants were significantly older than those who fit the model [26]. The age of the patient sample for the FreKAQ-J (68.5±9.1 years) was older than that of the FreBAQ-J (56.0±16.4 years), which might influence differences in the results of misfitting participants. Samples that include many younger participants with knee OA might return different results, though this is unlikely to be important as knee OA is overwhelmingly a problem in older age groups.

The average person endorsability of the FreKAQ-J (-0.92 logits) was consistent with the FreBAQ-J (-0.88 logits). The FreKAQ-J covered average and high levels of distorted body perception as well as the FreBAQ-J does and is best suited for use with people who experience frequent perceptual impairments. From a psychometric perspective, the FreKAQ-J could be improved by adding the scale items which may be more easily endorsed. However, the clinical value of this is unclear because low levels of impaired self-perception are unlikely to impact on clinical status.

Unidimensionality is an important prerequisite for summating any set of items [56–58]. Our study showed that the total score exhibited unidimensionality, indicating that distorted body perception, comprising three dimensions (neglect symptoms, reduced proprioceptive acuity, and body perception), in people with knee OA, is a singular construct. Therefore, a meaningful comparison of distorted body perception across patients should be conducted using the FreKAQ-J.

The performance of the FreKAQ-J has drawbacks pertaining to our study population. Item 7 and 9 exhibited an excessive positive misfit. Item 7 (“I feel like my knee is bigger (swollen)”) of the FreKAQ-J (-0.29 logits) was easier to endorse than that of the FreBAQ-J (0.67 logits). In item 7, participants were asked whether their knee felt more enlarged (swollen) than it really is. However, participants may respond on the basis of whether their knee was swollen, not considering that the item is asking about a difference between perception and reality. We did not examine whether the knee was swollen using MRI or ultrasound and no formal clinical measure was taken. If their knee felt more enlarged than it really is, it was considered to be an important clinical symptom pertaining to distorted body perception. Therefore, further research is required to investigate impairment in accurate knee-size estimation. Item 9 (“My knee feels differences with right and left (One side feels dull or fat)”) was the easiest to endorse in the FreKAQ-J (-1.21 logits) as well as FreBAQ-J (-1.02 logits). Item 9 of the FreKAQ-J exhibited a slightly excessive positive misfit, but did not exhibit misfit for the FreBAQ-J. In the development process, the word “leaning” in item 9 of FreKAQ-J was deleted because the knee does not experience “leaning,” unlike the low back. This difference could lead to excessive misfit. These two misfit items may have to be eliminated. On the other hand, despite the two misfit items (Item 7 and 9), the FreKAQ-J showed acceptable psychometric properties, including good internal consistency, validity of the questionnaire, unidimensionality, good test–retest reliability, and no DIF. Therefore, it should be carefully considered whether these two items are eliminated.

The noticeable difference in the FreKAQ-J total score between the knee OA group (mean 12.4) and the control group (mean 3.4) and the association with pain intensity, disability, pain catastrophization, kinesiophobia, and anxiety, support the discriminative and construct validity of the questionnaire. The data do support previous suggestions that body perception disturbance is related to poor outcomes [59], and psychological distress [60]. On the other hand, the current study did not show a relationship between body perception disturbance and depression. FreBAQ scores were related to depression in people with back pain [25] but our correction for multiple measures may have reduced the power of our study to detect it in people with chronic knee pain. Moreover, the present study did not show a relationship between body perception disturbance and pain duration. This suggests that clinical factors other than time are more important in driving disruption of self perception.

A major strength of this study is the Rasch analysis, which allowed a critical psychometric analysis in addition to validation analysis. Second, the testing for invariance by group membership, including gender, age, pain intensity, pain duration, or disability, is also a key aspect of evaluation, i.e., the absence of DIF provides confirmation during the comparison between groups. Next, the sample from three centers pertaining to inpatient and/or outpatient was collected. This increased the generalizability of the results for other people with knee OA.

A few limitations of the present study warrant consideration. First, our study included a relatively small sample size, which resulted in standard errors of Rasch measurements that are larger than those likely to be observed with a larger sample size; therefore, a second study with more subjects is needed to arrive at a definite conclusion. Second, this study did not include follow-up; therefore, features such as sensitivity to change and predictive validity were not examined.

## Conclusion

We developed FreKAQ-J by modifying FreBAQ-J. The FreKAQ-J fits the Rasch measurement model well and is suitable for use in people with knee OA. Altered body perception was significantly related to clinical status in people with knee OA and may be worth evaluating when managing people with knee OA.
